# Correction: Social vulnerability during the COVID-19 pandemic in Peru

**DOI:** 10.1371/journal.pgph.0003399

**Published:** 2024-06-13

**Authors:** Carlos Orlando Zegarra Zamalloa, Pavel J. Contreras, Laura R. Orellana, Pedro Antonio Riega Lopez, Shailendra Prasad, María Sofía Cuba Fuentes

The following information is missing from the Funding statement: LRO is sponsored by Emerge, the Emerging Diseases Epidemiology Research Training grant D43 TW007393 awarded by the Fogarty International Center of the US National Institutes of Health.
