# Supplementary material for: Social vulnerability during the COVID-19 pandemic in Peru
Source: PLOS Glob Public Health. 2022 Dec 2;2(12):e0001330. doi: 10.1371/journal.pgph.0001330 (PMC10021250; doi:10.1371/journal.pgph.0001330)
Supplement: Attachment — Submitted filename: Response to Reviewers.docx [file pgph.0001330.s001.docx]

Dear Executive Director,

We send the corrections to each observation made by the reviewers.

Reviewer #1: Nice manuscript.

Authors need to take note of the following:

**1. The abstract does not well represent the body of the work.**

We have paraphrased the abstract to provide the most relevant information about the study.

**2. Line 68: remove the underscore sign**

We have made the change and paraphrased it.

**3. Line 70-71: the sentence is incomplete**

We paraphrase and complete the sentence.

**4. Line 272-274: the citation appears counterintuitive. Are the authors sure of the information presented? Please verify**

We have added some other factors that could explain the results.

**5. Can the 33 variables be presented in tabular format for the sake of clarity?**

The variables are presented in tabular format in the results section (Table 1).

**6. Please provide justification for the choice of study variables.**

We have added epidemiological factors such as obesity, cardiovascular disease, and immunosuppression. These chronic diseases were directly related to a greater probability of severe forms and mortality due to COVID19 in general population. Likewise, these diseases add a component of social vulnerability in developing countries such as Peru, where a significant part of the population does not have effective access to healthcare services and, consequently, increase the socioeconomic vulnerability of the individual and the family (greater expenditure on health services and medicines, greater risk of poverty, unemployment).

In the case of health indicators, the evolution of the pandemic in countries such as Peru showed that the impact of COVID-19 on the population was strongly related to a weak health system unable to meet the basic needs of the population, unlike in developed countries, where the SVI does not consider these variables, since it is assumed that medical services can respond to emergencies. In many cases, there was a lack of Intensive Care beds for serious cases, specialists in Intensive Care, and other health professionals. For this reason, we decided to consider those variables related to the health system (material and human resources, above all) in the analysis.

Reviewer #2: This study aims to investigate the relationship between SVI-COVID factors and COVID mortality in 196 provinces in Peru. My comments/questions are listed below. Overall, the manuscript does not meet the standard of publications and requires substantial revision.

**1. I feel that Table 1 about SVI uses in Introduction is not needed and many are not relevant to this manuscript. The author may want to consider summarizing SVI in text.**

We placed the information in a paragraph, and the table was removed.

**2. Some sentences in Introduction do not provide meaningful messages. More in-depth literature review may be necessary. For example, page 6, line 112-113. However, SVI is expected to be different across provinces in any country. The extent of difference may be large or small. Social determinants are known to affect people and communities (this is very broad).**

We corrected the presentation of ideas and paraphrase the paragraph. We placed the information in a paragraph, and the table was removed.

**3. Please provide more details in data sources, in addition to listing the websites. For example, number of provinces (regions in Peru), brief summary of data.**

We add information about data sources and websites.

**For the mortality data, why was April 2020 to Sep 2021 selected?**

Information on the reason for the study period was added.

**Please also describe definition and approaches to identify death from COVID-19 in this section. I’m wondering if there is any difference in mortality over time?**

We add the definition and approaches to identify COVID-19 death.

**4. Page 7, line 141. The reference is not related to COVID-19. Suggest cite the reference right after SVI.**

We have corrected the place of the reference.

**5. In terms of estimation of SVI-COVID19, the variables were treated with equal weight. This may not be the most efficient approach. Ranking may result in some loss of information.**

It’s right that when converting into rankings, all the variables will have the same weight, but we maintain this position, as this is what was proposed by the CDC to elaborate the Social Vulnerability Index.

**6. Did the author mean to say random “intercept”? From my understanding, there were a total of 196 observations (provinces). Random intercept is not feasible in this case. What does “multiple analyses” mean? Replications or change of methods? Please list any covariates here as well.**

The random intercept model was used to consider the spatial autocorrelation between SVI-COVID-19 and outcome; however, with only 196 observations, we accept the observation. We modified the statistical model to a Negative Binomial Regression.

We refer by multiple analyses to the final model that adds the covariate population density. We have changed the name from multiple analysis to multivariate analysis.

**7. Please spell out all abbreviations the first time using it, e.g., CI, RR, IRRa. IRRa was used in text, whereas RR was used in Table 3. I think they refer to the same numbers? Please confirm what statistics to report and be consistent.**

The Negative Binomial Regression model used gives us the estimate of the crude and adjusted incidence rate ratio (IRR). It was modified in the text and Table 3.

**8. In table 2, should most of the numbers multiply by 100 since many units are in %? Please present lower and upper quartiles if choosing to report median and SD in the table.**

We agree; we modify table 2 according to this recommendation.

**9. In Discussion, the authors said that they adapted a validated tool to include COVID-19 specific factors to categorize the social vulnerability in various provinces of Peru (page 13, line 242). I don’t see where a validated tool was mentioned. Does this mean SVI? I suggest to say SVI directly. What are COVID-10 specific factors? Perhaps this means COVID-19 labs? Please clarify.**

In the first paragraph of the discussion section, we added the requested information on the validation of the instrument and on the denomination of SVI.

Likewise, we send the actions required about the tables, figures, and data.

1. Preparation of the map: The cartography of the base map of Peru was obtained from the website of the National Geographic Institute of Peru (<https://www.idep.gob.pe/>) the metadata is publicly accessible. Then with the results obtained in our research we designed the maps in the ArcGIS program.

The final ArcGIS file for each figure was uploaded to the github repository, <https://github.com/lorellanac/SVI-and-COVID-19-in-Peru.git>

Short links of each figure

Figure 1: t.ly/Roxu

Figure 2: t.ly/7zUu

Figure 3: t.ly/71jY

Figure 4: t.ly/YzwF

Figure 5: t.ly/rGtW

Figure 6: t.ly/FaDn

Figure 7: t.ly/IvsA

Additionally, in the repository description we attached the Creative Commons Attribution License (<https://creativecommons.org/licenses/by/4.0/>)

2. Please amend your detailed online Financial Disclosure statement. This is published with the article. It must therefore be completed in full sentences and contain the exact wording you wish to be published.
   a. State the initials, alongside each funding source, of each author to receive each grant.
      b. State what role the funders took in the study. If the funders had no role in your study, please state: “The funders had no role in study design, data collection and analysis, decision to publish, or preparation of the manuscript.”
3. Please ensure that the funders and grant numbers match between the Financial Disclosure field and the Funding Information tab in your submission form. Note that the funders must be provided in the same order in both places as well.
4. Please send a completed 'Competing Interests' statement, including any COIs declared by your co-authors. If you have no competing interests to declare, please state “The authors have declared that no competing interests exist”. Otherwise please declare all competing interests beginning with the statement “I have read the journal's policy and the authors of this manuscript have the following competing interests:”
5. We have already configured the link to be public access, our apologies <https://github.com/lorellanac/SVI-and-COVID-19-in-Peru.git>

We thank you for your recommendations and we hope to receive news from you soon.

Yours sincerely,

Pavel J. Contreras, MD.
